# Upper cervical intramedullary spinal metastasis of ovarian carcinoma: a case report and review of the literature

**DOI:** 10.1186/1752-1947-5-311

**Published:** 2011-07-14

**Authors:** Amrendra S Miranpuri, Sharad Rajpal, M Shahriar Salamat, John S Kuo

**Affiliations:** 1 Department of Neurological Surgery, School of Medicine and Public Health, University of Wisconsin, Madison, WI, USA; 2 Department of Pathology and Laboratory Medicine, School of Medicine and Public Health, University of Wisconsin, Madison, WI, USA

## Abstract

**Introduction:**

Currently there is no generalized approach to treating patients with intra-medullary spinal metastasis. High cervical spinal cord lesions can be particularly challenging cases, and may even be considered inoperable by some.

**Case report:**

We present what is, to the best of our knowledge, the first reported case of ovarian carcinoma (managed primarily with surgery) in a 65-year-old Caucasian woman metastasizing to the upper cervical spinal cord; we also review the relevant literature and discuss management strategies.

**Conclusions:**

Due to improving systemic cancer therapies, patients with cancer now often survive longer and are more likely to develop central nervous system metastases. Therefore, neurosurgical oncologists are often challenged with difficult decisions about how to surgically manage these patients. We recommend individualized multidisciplinary management based on patient functional status, the need for definitive diagnosis for possible additional adjuvant therapies, and consideration of extent of systemic disease impacting on desirable quality and length of survival.

## Introduction

Whereas lung and breast cancer represent the most frequently occurring spinal intra-medullary metastatic neoplasms, other solid tumors such as ovarian carcinoma can also rarely metastasize to the spinal cord. On imaging studies, the differential diagnoses for intra-medullary spinal lesions can include gliomas and vascular malformations but rare spinal infections such as tuberculosis can still be seen in some parts of the world [1,2]. The clinical presentation can range from minor neurological symptoms to major symptoms that significantly alter a patient's daily activities.

Surgical resection of intramedullary spinal metastases can be associated with significant morbidity. Management must therefore be individualized based on patient functional status, need for definitive diagnosis to guide additional therapies, and extent of systemic disease impacting on quality and length of survival. Previous reports have described management strategies for ovarian metastases to the spinal cord. However, we describe the first ever report of a high cervical ovarian metastasis managed primarily with surgery. Such an operation has potential airway and brainstem complications. As patients with cancer are surviving their primary disease longer, neurosurgical oncologists may be faced with the challenge of treating what were traditionally believed to be inoperable lesions. A careful discussion with the patient and their family, combined with multidisciplinary input from colleagues from medical and radiation oncology are important.

## Case presentation

A 65-year-old Caucasian woman underwent surgery for papillary serous ovarian adenocarcinoma involving both ovaries and with extensive metastases (stage IIIC). An exploratory laparotomy with total abdominal hysterectomy, bilateral salpingo-oophorectomy, and omentectomy with cancer staging was performed. She also underwent chemotherapy including carboplatin, paclitaxel, and cisplatin. Her CA-125 level was normal and there was no evidence of disease progression at her last clinic visit at our center. Then, two years later, she re-presented with progressive neurological symptoms starting initially with limb dysesthesias and numbness and progressing to quadriparesis with urinary retention.

**Figure 1:**
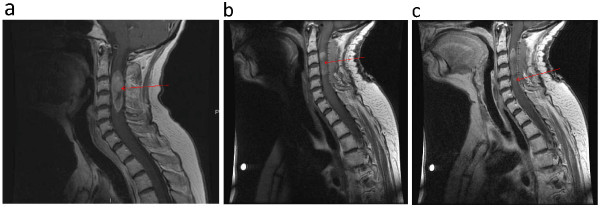
**Sagittal cervical spine MRI**. **(a) **Pre-surgical resection, T1 post-contrast demonstrating a 1.3 × 4.4 cm intramedullary enhancing mass (left panel). **(b) **Post-surgical resection, T1 pre-contrast (middle panel). **(c) **Post-surgical resection, T1 post-contrast showing small amount of residual tumor at caudal margin of tumor (right panel).

Imaging studies of her spine revealed an enhancing heterogeneous C2-C5 intramedullary lesion with cord expansion and edema extending rostrally into the medulla and caudally to the thoracic spinal cord (Figure 1a). Serum CA-125 was normal at presentation and a computed tomography (CT) scan of the chest, abdomen, and pelvis were negative for other lesions. An investigation for possible sources of infection was negative.

**Figure 2:**
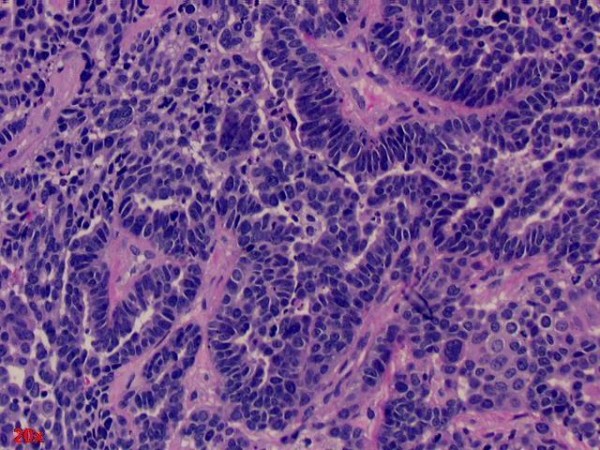
**Hematoxylin and eosin stained section of cervical intramedullary tumor**. This metastatic neoplasm was compared with prior hysterectomy and salpingo-oophorectomy of our patient and reveals similar histologic and cytologic features to the ovarian papillary serous adenocarcinoma.

Informed consent was obtained from our patient for open surgical biopsy and possible debulking. C2-C5 laminectomies were performed for planned ultrasound-guided dorsal midline biopsy and debulking of the intramedullary mass. The tumor was debulked and the remnants of the tumor capsule dissected along the rostral and caudal margins with care taken not to injure the surrounding spinal cord. Somatosensory and motor evoked potentials did not change during surgery. A post-operative MRI scan showed the expected near total resection and expected post-laminectomy changes without any associated hematoma (Figure 1b,c). Pathologic analysis revealed histological and cytological features consistent with papillary serous ovarian adenocarcinoma (Figure 2), similar to the pathological specimen from her prior surgery. She made functional improvements after surgery and was transferred to the rehabilitation service. She gained the ability to stand with assistance using a walker, had antigravity strength in her lower extremities and 4/5 strength in her upper extremities. Fractionated radiotherapy was initiated immediately in the post-operative period during her rehabilitation.

Our patient had improvement in strength post-operatively but required an emergency re-operation three weeks later due to sudden paraplegia secondary to spinal epidural hematoma, after therapy with prophylactic subcutaneous heparin administration. On discharge a week after epidural hematoma evacuation, she experienced numbness below the umbilicus and slightly improved to left toe movement. Unfortunately, our patient died five months after discovery of her spinal metastasis, presumably from a pulmonary embolism.

## Discussion

Review of the English language literature via PubMed database searches revealed five previous case reports of spinal cord ovarian cancer metastases, of which only three were tissue confirmed. Data from our report and the literature are summarized in Table 1.

**Table 1:** Summary of case reports published for intramedullary ovarian spinal tumors

Reference	Lesion level (enhancing portion)	Time from primary diagnosis to spinal metastasis diagnosis*	Surgical intervention	Adjuvant therapy	Outcome
Current report	C2-C5	two years	Subtotal resection	30Gy and steroids	Strength improved; three weeks post-operative spinal epidural hematoma; died five months later

Thomas *et al. *[6]	C6-T1	Four and a half years	None	30Gy and steroids	Strength improved; died six months later

Cormio *et al. *[8]	C5-C6	One and a half years	None	Steroids, chemotherapy, 30Gy	Strength improved; died 10 months later

Isoya *et al. *[5]	T10	four years	Subtotal resection	Radiotherapy (dose not given)	No neurological improvement; alive two years after surgery

Rastelli *et al. *[4]	T11	two years	Gross total resection	30Gy (10 fractions)	Near-complete strength improvement; MRI shows no spinal recurrence 16 months on

Bakshi *et al. *[7]	Conus medullaris and cauda equina	two years	None	Steroids, radiotherapy (dose not given), chemotherapy	Symptomatic improvement; three-year complete remission

*Approximate time in some cases based on estimates provided in reference.

Our patient's case is only the third reported tissue-proven case of ovarian carcinoma metastasizing to the spinal cord and the first reported case of metastasis to the high cervical spinal cord. Historically, there has been a role for surgery in resecting a solitary metastatic lesion to the spinal cord. The limitations to surgical resection are guided by the risk of morbidity to the patient, especially with regards to neurological function. Sundaresan *et al. *[3] retrospectively reviewed 80 patients with solitary spinal metastasis from all cancer histologies. Overall median survival in that series following surgery was 30 months. Survival was superior in the group with breast and kidney cancers. Morbidity and recurrence, however, were higher in patients receiving prior radiation therapy. Indications for surgery include pathological diagnosis, restoration of neurological function via decompression of mass effect and spinal stabilization [3].

The degree of tumor resection must be individualized. Rastelli *et al. *[4] reported gross total resection in a T11 metastatic ovarian cancer. This patient had near-complete strength improvement and MRI showed no spinal recurrence 16 months later. Even in the two cases of subtotal resection reviewed, tissue diagnosis is achieved while also achieving a less morbid operation as deemed appropriate by the involved surgeon. Isoyo *et al. *[5] performed a subtotal resection of a T10 metastatic ovarian lesion. This patient had no improvement in neurological status but remained alive two years after surgery.

Steroids are beneficial because it provides symptomatic relief and reduces peri-tumoral edema with a low side effect profile. The other case reports reviewed also described a 30Gy radiation dose as a preferred prescription for ovarian cancer spinal cord metastases. In select cases, simultaneous steroids and radiotherapy administration without a tissue diagnosis can be considered for patients at high surgical risk with poor Karnovsky scores [6,7]. Cormio *et al. *[8] demonstrated complete resolution of neurological symptoms with early steroids and carboplatin. Prior to the fourth cycle of carboplatin, an MRI scan of the brain showed diffuse metastatic disease for which the patient received 30Gy radiotherapy to the brain and cervical spine. In the above case report, however, no tissue diagnosis confirmation was obtained. The MRI scan performed after radiotherapy demonstrated almost complete resolution of the cervical lesion. Thus, steroids combined with chemotherapy and radiotherapy can be a viable empiric, alternative treatment regimen in high-risk surgical patients. Symptomatic and imaging responses in such cases, however, do not establish the diagnosis of ovarian spinal metastasis.

## Conclusions

There is no current consensus on management of patients presenting with neurological symptoms and a potential diagnosis of spinal intramedullary metastasis. In cases of central nervous system spinal cord metastases in patients experiencing progressive neurological symptoms whose medical condition permit surgery, we advocate open surgical biopsy with resection to confirm tissue diagnosis, to reduce tumor burden for adjuvant therapies while minimizing surgical morbidity, and to accurately diagnose and treat non-metastatic diseases that may masquerade as intramedullary spinal metastases. The risks and benefits of such interventions, however, must be carefully weighed in discussions with individual patients and their families. As patients with cancer are surviving their primary disease longer, it will be critical for neurosurgical oncologists to work closely with radiation oncologists and medical oncologists to formulate individualized treatment plans for patients with central nervous system metastases, based on risk/benefit analysis while also considering a patient's desire for quality of life and potential extent of survival.

## Consent

Written informed consent was obtained from the patient's next-of-kin for publication of this case report and any accompanying images. A copy of the written consent is available for review by the Editor-in-Chief of this journal.

## Competing interests

The authors declare that they have no competing interests.

## Authors' contributions

ASM, SR and JSK analyzed and interpreted our patient data regarding the clinical course, surgery and outcome. MSS performed the histological examination of the tumor. ASM was a major contributor in writing the manuscript. All authors read and approved the final manuscript.
